# Crystal structure of (3*E*,5*E*)-3,5-bis­[4-(di­ethyl­aza­nium­yl)benzyl­idene]-1-methyl-4-oxopiperidin-1-ium trichloride dihydrate: a potential biophotonic material

**DOI:** 10.1107/S2056989015020952

**Published:** 2015-11-21

**Authors:** Volodymyr V. Nesterov, Lev N. Zakharov, Vladimir N. Nesterov, Vladimir Shulaev

**Affiliations:** a Department of Chemistry, University of North Texas, Denton, TX 76203, USA; b CAMCOR Center for Advanced Materials Characterization in Oregon, University of Oregon, Eugene, Oregon 97403-1443, USA; c Department of Biological Sciences, University of North Texas, Denton, TX 76203, USA

**Keywords:** crystal structure, X-ray analysis, piperidinium salt, hydrogen bonding, biophotonic material

## Abstract

In the trication of the title hydrated mol­ecular salt, C_28_H_40_N_3_O^3+^·3Cl^−^·2H_2_O, the central heterocyclic ring adopts a sofa conformation, with the exocyclic N—C bond in an equatorial orientation. The dihedral angles between the planar part of this heterocyclic ring and the two almost flat side-chain fragments, which include the aromatic ring and bridging atoms, are 28.8 (1) and 41.1 (1)°. Both di­ethyl­aza­niumyl substituents have a tetra­hedral geometry, while the dihedral angles between the above-mentioned flat part of the aryl fragments and the imaginary planes drawn through atoms C—N—C of the di­ethyl­aza­niumyl substituents are 86.3 (2) and 80.4 (1)°, respectively. In the crystal, N—H⋯Cl hydrogen bonds link the cations and anions into [100] chains. The chains are cross-linked by numerous C—H⋯O and C—H⋯Cl inter­actions, generating a three-dimensional network. One of the chloride ions is disordered over two adjacent positions in a 0.895 (4):0.105 (4) ratio.

## Abstract 2

A stable oxopiperidinium trication salt was synthesized. In the crystal, N—H⋯Cl, C—H⋯Cl and C—H⋯O hydrogen bonds link cations and anions into a three-dimensional network.

## Chemical context   

In a continuation of our work on the synthesis and structural investigations of non-linear optical organic compounds with two-photon absorption properties and potential biophotonic materials (Nesterov *et al.*, 2003 ▸, 2007 ▸; Nesterov *et al.*, 2011*a* ▸,*b* ▸; Sarkisov *et al.*, 2005 ▸), we determined the crystal structure of the title compound. This compound belongs to a group that has shown anti­cancer activity (Jia *et al.*, 1988 ▸; Dimmock *et al.*, 2001 ▸). It may also find application as an agent for locating cancer cells with two-photon excited fluorescence and as a potential agent for a photodynamic treatment of cancer (Nesterov *et al.*, 2003 ▸; Sarkisov *et al.*, 2005 ▸).
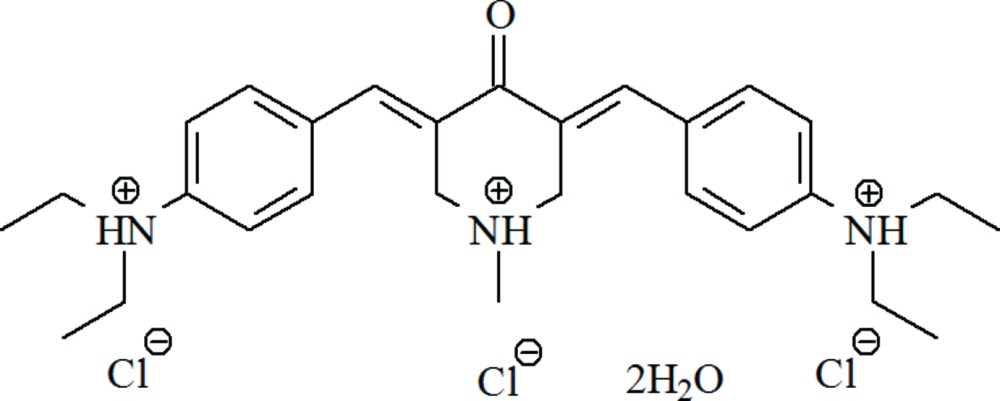


## Structural commentary   

**Figure 1:**
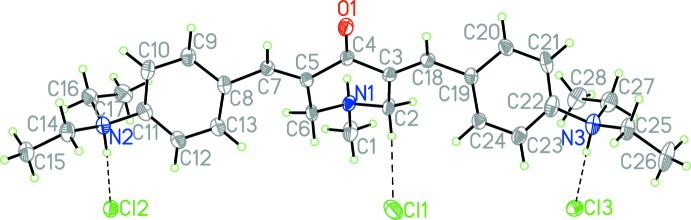
Perspective view of the trication and anions of (I), with hydrogen bonds shown as dashed lines. Displacement ellipsoids are drawn at the 30% probability level.

The structure of the trication with chloride anions is illustrated in Fig. 1 ▸. There are also two water mol­ecules of crystallization. The central heterocycle adopts a sofa conformation: atom N1 lies −0.732 (3) Å out of the central C_5_ plane [planar within 0.027 (2) Å]. The dihedral angles between the flat part of the heterocycle (atoms C2, C3, C4, C5, and C6) and the two almost planar fragments that include the phenyl-ring and the bridging atoms are 28.7 (1) and 41.1 (1)° for (C7–C13) and (C18–C24), respectively. Such non-planarity might partly be caused by the presence of short intra­molecular contacts H2*AB*⋯H24*A* and H6*AB*⋯H13*A* with distances 2.18 and 2.14 Å, respectively, which are shorter than the doubled van der Waals radius of the H atom (Rowland & Taylor, 1996 ▸). The mutual orientations of both aryl substit­uents relative to the flat part of the di­ethyl­aza­niumyl groups (N2, C14, C16 and N3, C25, C27) are almost orthogonal [dihedral angles of 86.3 (2) and 80.4 (1)°, respectively]. This is in contrast to the starting material where such angles are close to zero and the substituents participate in conjugated systems with the respective aromatic rings (Nesterov *et al.*, 2003 ▸).

## Supra­molecular features   

**Figure 2:**
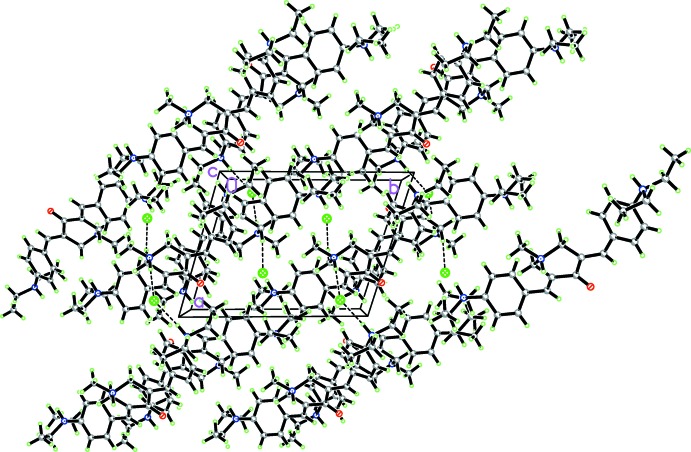
Projection of the crystal packing of the title compound along the *c* axis. Dashed lines denote strong inter­molecular N—H⋯Cl hydrogen bonds. Water mol­ecules have been omitted for clarity.

In the crystal, N—H⋯Cl hydrogen bonds (Table 1 ▸) link cations and anions (Fig. 2 ▸) into [100] chains. The chains are cross-linked by C—H⋯Cl and C—H⋯O inter­actions, forming a three-dimensional network. In addition, the existence of short (compared to the sum of the van der Waals radii of the corresponding pairs of atoms; Rowland & Taylor, 1996 ▸) inter­molecular water-to-water O⋯O and water-to-chloride O⋯Cl contacts presumably correspond to O—H⋯*X* hydrogen bonds, although the water H atoms could not be located in the present study.

## Database survey   

A search in the Cambridge Structural Database (Groom & Allen, 2014 ▸) for structures of piperidone with the amino substituents revealed eight hits with two salt structures of the oxopiperidinium iodide (Jia *et al.*, 1989 ▸; Nesterov *et al.*, 2007 ▸). Among these, there is a starting compound in which both di­ethyl­amino substituents participate in a conjugation with aromatic rings (Nesterov *et al.*, 2003 ▸).

## Synthesis and crystallization   

The starting compound (3*E*,5*E*)-3,5-bis­[4-(di­ethyl­amino)­benzyl­idene[−1-methyl-4-piperidone was obtained according to a literature procedure (Nesterov *et al.*, 2003 ▸). The relatively stable colorless crystals of the investigated salt were obtained by slow evaporation of the solution of the above piperidone from a mixture of ethanol and hydro­chloric acid over several days.

## Refinement   

Crystal data, data collection, and structure refinement details are summarized in Table 2 ▸. All C-bound H-atoms were placed in idealized positions and allowed to ride on their parent atom: C—H = 0.95, 0.99 and 0.98 Å for CH, CH_2_ and CH_3_ H atoms, respectively, with *U*_iso_(H) = k × *U*_eq_(C), where k = 1.2 for CH and CH_2_ and 1.5 for CH_3_ H atoms. All N-bound H atoms were located using difference Fourier maps, but in the final refinement their distances were constrained at 0.90 Å (DFIX). H atoms of the two water mol­ecules were not localized properly, since they appeared to be disordered over several positions. These H atoms were therefore removed from the refinement, but they were still included in the resulting chemical formula. Atom Cl3 is disordered over two positions in a 0.895 (4):0.105 (4) ratio.

## Supplementary Material

Crystal structure: contains datablock(s) I, global. DOI: 10.1107/S2056989015020952/hb7503sup1.cif

Structure factors: contains datablock(s) I. DOI: 10.1107/S2056989015020952/hb7503Isup2.hkl

Supporting information file. DOI: 10.1107/S2056989015020952/hb7503Isup3.cml

CCDC reference: 1435161

Additional supporting information:  crystallographic information; 3D view; checkCIF report

## Figures and Tables

**Table 1:** Hydrogen-bond geometry (Å, °)

*D*—H⋯*A*	*D*—H	H⋯*A*	*D*⋯*A*	*D*—H⋯*A*
N2—H2*C*⋯Cl2	0.91 (1)	2.27 (1)	3.166 (2)	169 (3)
N3—H3*A*⋯Cl3	0.91 (1)	2.14 (1)	3.054 (3)	175 (5)
N1—H1*D*⋯Cl2^i^	0.90 (1)	2.15 (1)	3.030 (2)	167 (2)
C1—H1*B*⋯Cl3^ii^	0.98	2.81	3.717 (4)	154
C2—H2*B*⋯Cl1	0.99	2.47	3.456 (3)	174
C6—H6*B*⋯Cl3^ii^	0.99	2.73	3.668 (3)	158
C10—H10*A*⋯O1*A* ^iii^	0.95	2.56	3.491 (4)	166
C16—H16*A*⋯Cl3^iii^	0.99	2.73	3.576 (3)	143
C20—H20*A*⋯O1^iv^	0.95	2.49	3.224 (3)	134
C21—H21*A*⋯Cl1^i^	0.95	2.68	3.602 (3)	164
C25—H25*A*⋯O1*A* ^v^	0.99	2.45	3.435 (4)	171
C27—H27*A*⋯Cl2^ii^	0.99	2.74	3.576 (3)	143
C27—H27*B*⋯Cl1^i^	0.99	2.66	3.621 (3)	164

Symmetry codes: (i) 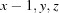; (ii) 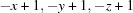; (iii) 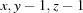; (iv) 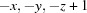; (v) 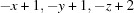.

**Table 2:** Experimental details

Crystal data	
Chemical formula	C_28_H_40_N_3_O^3+^·3Cl^−^·2H_2_O
*M*_r_	577.01
Crystal system, space group	Triclinic, *P* 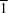
Temperature (K)	100
*a*, *b*, *c* (Å)	10.0933 (5), 12.0661 (6), 13.7576 (6)
α, β, γ (°)	97.759 (1), 110.795 (1), 102.733 (1)
*V* (Å^3^)	1485.46 (12)
*Z*	2
Radiation type	Mo *K*α
μ (mm^−1^)	0.34
Crystal size (mm)	0.18 × 0.12 × 0.10

Data collection	
Diffractometer	Bruker APEXII CCD
Absorption correction	Multi-scan (*SADABS*; Bruker, 2007 ▸)
*T*_min_, *T*_max_	0.941, 0.967
No. of measured, independent and observed [*I* > 2σ(*I*)] reflections	11768, 5787, 5056
*R*_int_	0.016
(sin θ/λ)_max_ (Å^−1^)	0.617

Refinement	
*R*[*F* ^2^ > 2σ(*F* ^2^)], *wR*(*F* ^2^), *S*	0.058, 0.164, 1.05
No. of reflections	5787
No. of parameters	356
No. of restraints	3
H-atom treatment	H atoms treated by a mixture of independent and constrained refinement
Δρ_max_, Δρ_min_ (e Å^−3^)	1.13, −0.62

Computer programs: *APEX2* and *SAINT* (Bruker, 2007 ▸), *SHELXS97*, *SHELXL97* and *SHELXTL* (Sheldrick, 2008 ▸).

## supplementary crystallographic information

### Crystal data

**Table:**

C_28_H_40_N_3_O^3+^·3Cl^−^·2H_2_O	*Z* = 2
*M**_r_* = 577.01	*F*(000) = 616
Triclinic, *P*1	*D*_x_ = 1.290 Mg m^−^^3^
Hall symbol: -P 1	Mo *K*α radiation, λ = 0.71073 Å
*a* = 10.0933 (5) Å	Cell parameters from 4567 reflections
*b* = 12.0661 (6) Å	θ = 2.5–26.0°
*c* = 13.7576 (6) Å	µ = 0.34 mm^−^^1^
α = 97.759 (1)°	*T* = 100 K
β = 110.795 (1)°	Block, colourless
γ = 102.733 (1)°	0.18 × 0.12 × 0.10 mm
*V* = 1485.46 (12) Å^3^	

### Data collection

**Table:**

Bruker APEXII CCD diffractometer	5787 independent reflections
Radiation source: fine-focus sealed tube	5056 reflections with *I* > 2σ(*I*)
Graphite monochromator	*R*_int_ = 0.016
φ and ω scans	θ_max_ = 26.0°, θ_min_ = 1.6°
Absorption correction: multi-scan (*SADABS*; Bruker, 2007)	*h* = −12→12
*T*_min_ = 0.941, *T*_max_ = 0.967	*k* = −14→14
11768 measured reflections	*l* = −16→16

### Refinement

**Table:**

Refinement on *F*^2^	Primary atom site location: structure-invariant direct methods
Least-squares matrix: full	Secondary atom site location: difference Fourier map
*R*[*F*^2^ > 2σ(*F*^2^)] = 0.058	Hydrogen site location: inferred from neighbouring sites
*wR*(*F*^2^) = 0.164	H atoms treated by a mixture of independent and constrained refinement
*S* = 1.05	*w* = 1/[σ^2^(*F*_o_^2^) + (0.091*P*)^2^ + 1.9*P*] where *P* = (*F*_o_^2^ + 2*F*_c_^2^)/3
5787 reflections	(Δ/σ)_max_ = 0.001
356 parameters	Δρ_max_ = 1.13 e Å^−^^3^
3 restraints	Δρ_min_ = −0.62 e Å^−^^3^

### Special details

**Table:**

Geometry. All esds (except the esd in the dihedral angle between two l.s. planes) are estimated using the full covariance matrix. The cell esds are taken into account individually in the estimation of esds in distances, angles and torsion angles; correlations between esds in cell parameters are only used when they are defined by crystal symmetry. An approximate (isotropic) treatment of cell esds is used for estimating esds involving l.s. planes.
Refinement. Refinement of F^2^ against ALL reflections. The weighted R-factor wR and goodness of fit S are based on F^2^, conventional R-factors R are based on F, with F set to zero for negative F^2^. The threshold expression of F^2^ > 2sigma(F^2^) is used only for calculating R-factors(gt) etc. and is not relevant to the choice of reflections for refinement. R-factors based on F^2^ are statistically about twice as large as those based on F, and R- factors based on ALL data will be even larger.

### Fractional atomic coordinates and isotropic or equivalent isotropic displacement parameters (Å^2^)

**Table:**

	*x*	*y*	*z*	*U*_iso_*/*U*_eq_	Occ. (<1)
Cl1	0.69912 (7)	0.36862 (6)	0.70014 (6)	0.0379 (2)	
Cl2	1.10763 (7)	0.16702 (5)	0.19807 (5)	0.02863 (18)	
Cl3	0.41255 (10)	0.74605 (6)	0.84491 (6)	0.0319 (3)	0.895 (4)
Cl3A	0.4816 (15)	0.7207 (9)	0.8555 (8)	0.059 (3)*	0.105 (4)
O1	0.2253 (2)	−0.04179 (15)	0.41664 (15)	0.0288 (4)	
N1	0.4116 (2)	0.26939 (17)	0.37806 (17)	0.0229 (4)	
H1D	0.3294 (19)	0.239 (2)	0.3181 (14)	0.017 (6)*	
N2	0.8498 (2)	−0.06652 (19)	0.13943 (17)	0.0251 (5)	
H2C	0.924 (3)	0.0015 (16)	0.165 (2)	0.034 (8)*	
N3	0.1387 (3)	0.5404 (2)	0.78810 (19)	0.0308 (5)	
H3A	0.221 (3)	0.602 (3)	0.809 (4)	0.093 (17)*	
C1	0.4892 (4)	0.3863 (2)	0.3704 (3)	0.0365 (7)	
H1A	0.4236	0.4366	0.3621	0.055*	
H1B	0.5157	0.3772	0.3084	0.055*	
H1C	0.5792	0.4220	0.4356	0.055*	
C2	0.3736 (3)	0.2804 (2)	0.4734 (2)	0.0237 (5)	
H2A	0.3144	0.3362	0.4704	0.028*	
H2B	0.4656	0.3114	0.5392	0.028*	
C3	0.2870 (3)	0.1637 (2)	0.47709 (18)	0.0204 (5)	
C4	0.3029 (3)	0.0544 (2)	0.42288 (19)	0.0213 (5)	
C5	0.4212 (3)	0.0674 (2)	0.37980 (18)	0.0204 (5)	
C6	0.5053 (3)	0.1887 (2)	0.3839 (2)	0.0249 (5)	
H6A	0.5953	0.2179	0.4513	0.030*	
H6B	0.5368	0.1866	0.3235	0.030*	
C7	0.4468 (3)	−0.0299 (2)	0.33955 (18)	0.0204 (5)	
H7A	0.3874	−0.1019	0.3414	0.024*	
C8	0.5559 (3)	−0.0379 (2)	0.29304 (19)	0.0223 (5)	
C9	0.5226 (3)	−0.1372 (2)	0.2139 (2)	0.0261 (5)	
H9A	0.4346	−0.1988	0.1951	0.031*	
C10	0.6172 (3)	−0.1468 (2)	0.1624 (2)	0.0299 (6)	
H10A	0.5930	−0.2134	0.1070	0.036*	
C11	0.7474 (3)	−0.0574 (2)	0.1932 (2)	0.0255 (5)	
C12	0.7871 (3)	0.0384 (2)	0.2743 (2)	0.0273 (5)	
H12A	0.8786	0.0970	0.2960	0.033*	
C13	0.6914 (3)	0.0482 (2)	0.3240 (2)	0.0274 (5)	
H13A	0.7179	0.1145	0.3803	0.033*	
C14	0.9212 (3)	−0.1619 (2)	0.1686 (2)	0.0294 (6)	
H14A	0.9628	−0.1503	0.2474	0.035*	
H14B	0.8442	−0.2384	0.1379	0.035*	
C15	1.0423 (3)	−0.1647 (3)	0.1296 (2)	0.0335 (6)	
H15A	1.0940	−0.2198	0.1599	0.050*	
H15B	1.1127	−0.0865	0.1521	0.050*	
H15C	0.9992	−0.1897	0.0514	0.050*	
C16	0.7779 (3)	−0.0775 (3)	0.0204 (2)	0.0314 (6)	
H16A	0.6993	−0.1530	−0.0129	0.038*	
H16B	0.8528	−0.0778	−0.0104	0.038*	
C17	0.7110 (3)	0.0207 (3)	−0.0065 (2)	0.0357 (6)	
H17A	0.6672	0.0103	−0.0844	0.054*	
H17B	0.7883	0.0957	0.0258	0.054*	
H17C	0.6340	0.0196	0.0214	0.054*	
C18	0.1975 (3)	0.1549 (2)	0.53023 (19)	0.0205 (5)	
H18A	0.1401	0.0785	0.5243	0.025*	
C19	0.1804 (3)	0.2522 (2)	0.59652 (19)	0.0203 (5)	
C20	0.0408 (3)	0.2538 (2)	0.5932 (2)	0.0247 (5)	
H20A	−0.0434	0.1902	0.5488	0.030*	
C21	0.0235 (3)	0.3478 (3)	0.6542 (2)	0.0300 (6)	
H21A	−0.0719	0.3494	0.6503	0.036*	
C22	0.1474 (3)	0.4382 (2)	0.7200 (2)	0.0285 (6)	
C23	0.2872 (3)	0.4361 (2)	0.7293 (2)	0.0276 (5)	
H23A	0.3716	0.4972	0.7780	0.033*	
C24	0.3036 (3)	0.3444 (2)	0.6671 (2)	0.0245 (5)	
H24A	0.3998	0.3437	0.6723	0.029*	
C25	0.1258 (3)	0.5097 (2)	0.8880 (2)	0.0312 (6)	
H25A	0.1994	0.4683	0.9191	0.037*	
H25B	0.0260	0.4562	0.8689	0.037*	
C26	0.1511 (5)	0.6178 (3)	0.9711 (3)	0.0505 (9)	
H26A	0.1669	0.5976	1.0402	0.076*	
H26B	0.0643	0.6469	0.9486	0.076*	
H26C	0.2385	0.6784	0.9779	0.076*	
C27	0.0234 (3)	0.5949 (2)	0.7310 (2)	0.0336 (6)	
H27A	0.0316	0.6662	0.7805	0.040*	
H27B	−0.0757	0.5397	0.7110	0.040*	
C28	0.0356 (4)	0.6268 (3)	0.6323 (2)	0.0416 (7)	
H28A	−0.0141	0.6870	0.6145	0.062*	
H28B	−0.0115	0.5574	0.5728	0.062*	
H28C	0.1403	0.6569	0.6451	0.062*	
O1A	0.5901 (3)	0.6022 (2)	0.9885 (3)	0.0693 (8)	
O2A	0.7711 (5)	0.5914 (3)	0.8786 (3)	0.0984 (12)	

### Atomic displacement parameters (Å^2^)

**Table:**

	*U*^11^	*U*^22^	*U*^33^	*U*^12^	*U*^13^	*U*^23^
Cl1	0.0277 (3)	0.0416 (4)	0.0486 (4)	0.0113 (3)	0.0192 (3)	0.0105 (3)
Cl2	0.0306 (3)	0.0305 (3)	0.0273 (3)	0.0124 (3)	0.0130 (3)	0.0049 (2)
Cl3	0.0304 (5)	0.0243 (4)	0.0365 (5)	0.0025 (3)	0.0142 (3)	−0.0012 (3)
O1	0.0339 (10)	0.0195 (9)	0.0368 (10)	0.0005 (7)	0.0247 (8)	0.0012 (7)
N1	0.0286 (11)	0.0174 (10)	0.0253 (11)	0.0029 (8)	0.0169 (9)	0.0023 (8)
N2	0.0276 (11)	0.0259 (11)	0.0264 (11)	0.0107 (9)	0.0140 (9)	0.0071 (9)
N3	0.0442 (14)	0.0291 (12)	0.0295 (12)	0.0167 (11)	0.0221 (11)	0.0090 (10)
C1	0.0512 (18)	0.0193 (12)	0.0517 (18)	0.0051 (12)	0.0380 (15)	0.0080 (12)
C2	0.0297 (13)	0.0205 (11)	0.0241 (12)	0.0045 (10)	0.0174 (10)	0.0016 (9)
C3	0.0209 (11)	0.0209 (11)	0.0190 (11)	0.0048 (9)	0.0089 (9)	0.0033 (9)
C4	0.0226 (11)	0.0223 (12)	0.0198 (11)	0.0040 (9)	0.0114 (10)	0.0029 (9)
C5	0.0200 (11)	0.0221 (11)	0.0202 (11)	0.0042 (9)	0.0109 (9)	0.0039 (9)
C6	0.0248 (12)	0.0208 (12)	0.0336 (13)	0.0047 (10)	0.0193 (11)	0.0020 (10)
C7	0.0213 (11)	0.0222 (11)	0.0190 (11)	0.0061 (9)	0.0091 (9)	0.0059 (9)
C8	0.0281 (12)	0.0247 (12)	0.0232 (12)	0.0141 (10)	0.0146 (10)	0.0114 (10)
C9	0.0306 (13)	0.0242 (12)	0.0304 (13)	0.0115 (10)	0.0168 (11)	0.0094 (10)
C10	0.0390 (15)	0.0258 (13)	0.0312 (14)	0.0159 (11)	0.0181 (12)	0.0049 (11)
C11	0.0277 (13)	0.0311 (13)	0.0272 (13)	0.0132 (11)	0.0160 (11)	0.0154 (11)
C12	0.0255 (12)	0.0276 (13)	0.0340 (14)	0.0094 (10)	0.0161 (11)	0.0090 (11)
C13	0.0268 (13)	0.0293 (13)	0.0314 (14)	0.0112 (11)	0.0156 (11)	0.0081 (11)
C14	0.0357 (14)	0.0277 (13)	0.0323 (14)	0.0166 (11)	0.0163 (12)	0.0106 (11)
C15	0.0347 (15)	0.0333 (14)	0.0391 (15)	0.0163 (12)	0.0185 (13)	0.0077 (12)
C16	0.0291 (13)	0.0477 (16)	0.0205 (13)	0.0132 (12)	0.0122 (11)	0.0078 (11)
C17	0.0347 (15)	0.0444 (16)	0.0331 (15)	0.0120 (13)	0.0170 (12)	0.0150 (13)
C18	0.0218 (11)	0.0189 (11)	0.0215 (11)	0.0045 (9)	0.0098 (9)	0.0052 (9)
C19	0.0261 (12)	0.0207 (11)	0.0208 (11)	0.0081 (9)	0.0147 (10)	0.0092 (9)
C20	0.0229 (12)	0.0305 (13)	0.0242 (12)	0.0071 (10)	0.0131 (10)	0.0087 (10)
C21	0.0350 (14)	0.0423 (15)	0.0314 (14)	0.0229 (12)	0.0241 (12)	0.0185 (12)
C22	0.0463 (16)	0.0261 (13)	0.0242 (12)	0.0179 (12)	0.0204 (12)	0.0115 (10)
C23	0.0367 (14)	0.0238 (12)	0.0247 (12)	0.0056 (11)	0.0170 (11)	0.0049 (10)
C24	0.0265 (12)	0.0253 (12)	0.0240 (12)	0.0051 (10)	0.0145 (10)	0.0050 (10)
C25	0.0481 (16)	0.0274 (13)	0.0302 (14)	0.0151 (12)	0.0256 (13)	0.0104 (11)
C26	0.093 (3)	0.0444 (18)	0.0347 (16)	0.0295 (19)	0.0425 (18)	0.0120 (14)
C27	0.0432 (16)	0.0266 (13)	0.0366 (15)	0.0180 (12)	0.0171 (13)	0.0087 (11)
C28	0.057 (2)	0.0377 (16)	0.0338 (16)	0.0221 (15)	0.0162 (14)	0.0130 (13)
O1A	0.0472 (14)	0.0392 (13)	0.104 (2)	0.0153 (11)	0.0153 (15)	−0.0023 (14)
O2A	0.142 (4)	0.075 (2)	0.076 (2)	0.027 (2)	0.044 (2)	0.0198 (18)

### Geometric parameters (Å, º)

**Table:**

O1—C4	1.221 (3)	C13—H13A	0.9500
N1—C2	1.489 (3)	C14—C15	1.504 (4)
N1—C6	1.491 (3)	C14—H14A	0.9900
N1—C1	1.492 (3)	C14—H14B	0.9900
N1—H1D	0.895 (10)	C15—H15A	0.9800
N2—C11	1.483 (3)	C15—H15B	0.9800
N2—C16	1.510 (3)	C15—H15C	0.9800
N2—C14	1.513 (3)	C16—C17	1.513 (4)
N2—H2C	0.907 (10)	C16—H16A	0.9900
N3—C22	1.483 (3)	C16—H16B	0.9900
N3—C27	1.494 (4)	C17—H17A	0.9800
N3—C25	1.512 (3)	C17—H17B	0.9800
N3—H3A	0.912 (10)	C17—H17C	0.9800
C1—H1A	0.9800	C18—C19	1.468 (3)
C1—H1B	0.9800	C18—H18A	0.9500
C1—H1C	0.9800	C19—C20	1.398 (3)
C2—C3	1.503 (3)	C19—C24	1.402 (4)
C2—H2A	0.9900	C20—C21	1.399 (4)
C2—H2B	0.9900	C20—H20A	0.9500
C3—C18	1.345 (3)	C21—C22	1.379 (4)
C3—C4	1.495 (3)	C21—H21A	0.9500
C4—C5	1.499 (3)	C22—C23	1.377 (4)
C5—C7	1.346 (3)	C23—C24	1.382 (4)
C5—C6	1.503 (3)	C23—H23A	0.9500
C6—H6A	0.9900	C24—H24A	0.9500
C6—H6B	0.9900	C25—C26	1.521 (4)
C7—C8	1.470 (3)	C25—H25A	0.9900
C7—H7A	0.9500	C25—H25B	0.9900
C8—C9	1.394 (4)	C26—H26A	0.9800
C8—C13	1.404 (4)	C26—H26B	0.9800
C9—C10	1.391 (4)	C26—H26C	0.9800
C9—H9A	0.9500	C27—C28	1.499 (4)
C10—C11	1.388 (4)	C27—H27A	0.9900
C10—H10A	0.9500	C27—H27B	0.9900
C11—C12	1.367 (4)	C28—H28A	0.9800
C12—C13	1.383 (4)	C28—H28B	0.9800
C12—H12A	0.9500	C28—H28C	0.9800
			
C2—N1—C6	109.88 (19)	N2—C14—H14A	108.9
C2—N1—C1	110.58 (19)	C15—C14—H14B	108.9
C6—N1—C1	110.6 (2)	N2—C14—H14B	108.9
C2—N1—H1D	110.5 (18)	H14A—C14—H14B	107.7
C6—N1—H1D	107.7 (17)	C14—C15—H15A	109.5
C1—N1—H1D	107.5 (17)	C14—C15—H15B	109.5
C11—N2—C16	112.58 (19)	H15A—C15—H15B	109.5
C11—N2—C14	110.55 (19)	C14—C15—H15C	109.5
C16—N2—C14	113.6 (2)	H15A—C15—H15C	109.5
C11—N2—H2C	108 (2)	H15B—C15—H15C	109.5
C16—N2—H2C	105 (2)	N2—C16—C17	112.6 (2)
C14—N2—H2C	107 (2)	N2—C16—H16A	109.1
C22—N3—C27	114.4 (2)	C17—C16—H16A	109.1
C22—N3—C25	110.3 (2)	N2—C16—H16B	109.1
C27—N3—C25	112.8 (2)	C17—C16—H16B	109.1
C22—N3—H3A	112 (3)	H16A—C16—H16B	107.8
C27—N3—H3A	99 (3)	C16—C17—H17A	109.5
C25—N3—H3A	108 (3)	C16—C17—H17B	109.5
N1—C1—H1A	109.5	H17A—C17—H17B	109.5
N1—C1—H1B	109.5	C16—C17—H17C	109.5
H1A—C1—H1B	109.5	H17A—C17—H17C	109.5
N1—C1—H1C	109.5	H17B—C17—H17C	109.5
H1A—C1—H1C	109.5	C3—C18—C19	126.0 (2)
H1B—C1—H1C	109.5	C3—C18—H18A	117.0
N1—C2—C3	110.49 (19)	C19—C18—H18A	117.0
N1—C2—H2A	109.6	C20—C19—C24	118.1 (2)
C3—C2—H2A	109.6	C20—C19—C18	120.7 (2)
N1—C2—H2B	109.6	C24—C19—C18	121.1 (2)
C3—C2—H2B	109.6	C19—C20—C21	120.9 (2)
H2A—C2—H2B	108.1	C19—C20—H20A	119.5
C18—C3—C4	118.8 (2)	C21—C20—H20A	119.5
C18—C3—C2	121.6 (2)	C22—C21—C20	118.7 (2)
C4—C3—C2	119.6 (2)	C22—C21—H21A	120.6
O1—C4—C3	121.3 (2)	C20—C21—H21A	120.6
O1—C4—C5	121.4 (2)	C23—C22—C21	121.6 (2)
C3—C4—C5	117.3 (2)	C23—C22—N3	116.2 (2)
C7—C5—C4	118.3 (2)	C21—C22—N3	122.1 (2)
C7—C5—C6	123.4 (2)	C22—C23—C24	119.4 (3)
C4—C5—C6	118.2 (2)	C22—C23—H23A	120.3
N1—C6—C5	110.69 (19)	C24—C23—H23A	120.3
N1—C6—H6A	109.5	C23—C24—C19	121.0 (2)
C5—C6—H6A	109.5	C23—C24—H24A	119.5
N1—C6—H6B	109.5	C19—C24—H24A	119.5
C5—C6—H6B	109.5	N3—C25—C26	111.9 (2)
H6A—C6—H6B	108.1	N3—C25—H25A	109.2
C5—C7—C8	127.6 (2)	C26—C25—H25A	109.2
C5—C7—H7A	116.2	N3—C25—H25B	109.2
C8—C7—H7A	116.2	C26—C25—H25B	109.2
C9—C8—C13	118.3 (2)	H25A—C25—H25B	107.9
C9—C8—C7	117.8 (2)	C25—C26—H26A	109.5
C13—C8—C7	123.8 (2)	C25—C26—H26B	109.5
C10—C9—C8	120.6 (2)	H26A—C26—H26B	109.5
C10—C9—H9A	119.7	C25—C26—H26C	109.5
C8—C9—H9A	119.7	H26A—C26—H26C	109.5
C11—C10—C9	118.9 (2)	H26B—C26—H26C	109.5
C11—C10—H10A	120.6	N3—C27—C28	113.5 (2)
C9—C10—H10A	120.6	N3—C27—H27A	108.9
C12—C11—C10	122.0 (2)	C28—C27—H27A	108.9
C12—C11—N2	118.4 (2)	N3—C27—H27B	108.9
C10—C11—N2	119.6 (2)	C28—C27—H27B	108.9
C11—C12—C13	118.7 (2)	H27A—C27—H27B	107.7
C11—C12—H12A	120.6	C27—C28—H28A	109.5
C13—C12—H12A	120.6	C27—C28—H28B	109.5
C12—C13—C8	121.3 (2)	H28A—C28—H28B	109.5
C12—C13—H13A	119.3	C27—C28—H28C	109.5
C8—C13—H13A	119.3	H28A—C28—H28C	109.5
C15—C14—N2	113.3 (2)	H28B—C28—H28C	109.5
C15—C14—H14A	108.9		

### Hydrogen-bond geometry (Å, º)

**Table:**

*D*—H···*A*	*D*—H	H···*A*	*D*···*A*	*D*—H···*A*
N2—H2*C*···Cl2	0.91 (1)	2.27 (1)	3.166 (2)	169 (3)
N3—H3*A*···Cl3	0.91 (1)	2.14 (1)	3.054 (3)	175 (5)
N1—H1*D*···Cl2^i^	0.90 (1)	2.15 (1)	3.030 (2)	167 (2)
C1—H1*B*···Cl3^ii^	0.98	2.81	3.717 (4)	154
C2—H2*B*···Cl1	0.99	2.47	3.456 (3)	174
C6—H6*B*···Cl3^ii^	0.99	2.73	3.668 (3)	158
C10—H10*A*···O1*A*^iii^	0.95	2.56	3.491 (4)	166
C16—H16*A*···Cl3^iii^	0.99	2.73	3.576 (3)	143
C20—H20*A*···O1^iv^	0.95	2.49	3.224 (3)	134
C21—H21*A*···Cl1^i^	0.95	2.68	3.602 (3)	164
C25—H25*A*···O1*A*^v^	0.99	2.45	3.435 (4)	171
C27—H27*A*···Cl2^ii^	0.99	2.74	3.576 (3)	143
C27—H27*B*···Cl1^i^	0.99	2.66	3.621 (3)	164

Symmetry codes: (i) *x*−1, *y*, *z*; (ii) −*x*+1, −*y*+1, −*z*+1; (iii) *x*, *y*−1, *z*−1; (iv) −*x*, −*y*, −*z*+1; (v) −*x*+1, −*y*+1, −*z*+2.
